# Insight into Y Content on Microstructure and Mechanical Properties of Mg-Gd-Y-Zr Alloy

**DOI:** 10.3390/ma18112475

**Published:** 2025-05-25

**Authors:** Yongfeng Li, Ang Zhang, Chuangming Li, Hecong Xie, Hengrui Hu, Yuyang Gao, Yuhong Cui, Zhihua Dong, Tian Li, Bin Jiang

**Affiliations:** 1 National Engineering Research Center for Magnesium Alloys, National Key Laboratory of Advanced Casting Technologies, College of Materials Science and Engineering, Chongqing University, Chongqing 400044, China; yongfengli9275@163.com (Y.L.); chuangming_li@163.com (C.L.); 17316748136@163.com (H.X.); huhenry@stu.cqu.edu.cn (H.H.); gaoyuyang@cqu.edu.cn (Y.G.); dzhihua@cqu.edu.cn (Z.D.); 2 Qinghai Salt Lake Teli Magnesium Co., Ltd., Xining 810000, China; www_yanbulishou@163.com; 3 Zhejiang Wanfeng Precision Casting Co., Ltd., Shaoxing 312000, China; tianli89@cqu.edu.cn

**Keywords:** Mg alloy, Y content, microstructure evolution, mechanical properties

## Abstract

This paper investigates the effects of Y content on the microstructure and mechanical properties of Mg-9Gd-*x*Y-0.5Zr alloys. The mechanical properties, together with the grain size, first increase and then decrease, exhibiting a non-monotonous change with the increase in Y content. The alloy with 3 wt.% Y exhibits the best mechanical properties compared to the Y free alloy, with an increase in ultimate tensile strength and yield strength of 33.4% and 19.2%, respectively. However, further increase in the Y content (5 wt.%) does not enhance the strength but promotes the growth of the Mg_5_(Gd,Y), becoming nucleation points and propagation paths for cracks, leading to a decrease in performance. The cubic phase REH_2_ and oxide are not the reasons for the poor ductility in as-cast Mg-9Gd-*x*Y-0.5Zr alloys due to low content, and the crystallographic orientation relationship between REH_2_ and α-Mg is (220)_REH2_//(11$\bar{2}$0)_Mg_, and the interface mismatch is 9.13%. This paper systematically prepares and investigates the high-strength as-cast Mg-Gd-Y-Zr alloy, which has important guiding significance.

## 1. Introduction

The use of magnesium (Mg) alloys in automobile and aircraft components has sparked intense interest due to low density, good electromagnetic shielding, and machining ability [1,2,3,4,5]. Widening the application of Mg alloys is crucial to alleviating the energy crisis and reducing carbon emissions [6,7,8,9]. However, Mg alloys have relatively low absolute strength and poor formability [10,11,12]. Extensive research and development efforts have been devoted to enhancing their mechanical strength [13,14,15], formability [16], and creep resistance [17] through utilizing strengthening theory such as grain refinement [18], aging hardening [19,20], composite strengthening [21,22], and texture strengthening [23].

In addition to the above ways, alloying is a widely used and proven technique to enhance the mechanical properties of Mg alloys [24]. Adding RE to Mg alloys can improve casting performance and high-temperature properties, increase fluidity, purify the melt, and produce stronger precipitation and dispersion strengthening effects [25,26]. Adding Y to Mg alloys can reduce the solid-liquid interface tension and the nucleation energy, and hinder grain growth by forming rich-Y phases with high melting points. Hu et al. [27] found that adding Y to the Mg-6Zn-1Mn-4Sn alloy can form the MgSnY phase, refine grains, and enhance comprehensive mechanical properties. Additionally, adding Zr can refine grains by increasing nucleation points of α-Mg [28]. Wei et al. [29] prepare Mg-8Gd-3Y-0.5Zr alloy by extrusion, predeformation, and aging; the synergistic effect of nano-precipitates and bimodal microstructure simultaneously enhances strength and ductility.

The as-cast Mg-Gd-Y-Zr alloys exhibit poor strength during room temperature deformation. The as-cast Mg-15Gd-5Y-0.5Zr alloy with a high rare earth (RE) content achieved UTS and YS of 247 MPa and 193 MPa, respectively, but the EL is only 0.8% [30]. Jiang et al. [31] prepare alloys such as GW133K, GW123K, GW103K, and GW93K using the steel mold of bottom gating type, and the optimal UTS, YS, and EL are 251 MPa, 209 MPa, and 0.47%, respectively. The sand-cast Mg-9Gd-4Y-0.5Zr exhibits lower UTS and EL, only reaching 200 MPa and 2.2%, respectively [32]. Although Mg-Gd-Y-Zr alloys have been extensively investigated; however, there is limited systematic research on the RE hydrides, oxidation tendencies, defects, and mechanical properties in as-cast Mg-Gd-Y-Zr alloys.

This paper will analyze the impact of Y content on Mg-Gd-Y-Zr alloys by delving into the relationships between the second phases, defects, and properties, providing ideas and guidance for the composition design, organizational optimization, and performance improvement. This paper systematically explains the influence mechanisms of Y addition on the strengthening and fracture mechanisms of as-cast Mg-Gd-Y-Zr alloys and reveals the impact of Y content on the microstructure and properties of as-cast Mg-Gd-Y-Zr alloys.

## 2. Experiments

The Mg-9Gd-*x*Y-0.5Zr (*x* = 0, 1, 3, 5 wt.%) alloys are prepared by melting pure Mg, Mg-30Gd, Mg-30Y, and Mg-30Zr master alloys in an electric resistance furnace under the protection of an atmosphere composed of SF_6_ (1 vol%) and CO_2_ (99 vol%). Mg-30Gd and Mg-30Y are added to the Mg melt at 740 °C. and Mg-Zr is added at 780 °C. After all the master alloys are melted, the melt is stirred for 5 min and then allowed to settle for 30 min, subsequently, cool the melt to 740 °C, and finally, the melt is poured into a mold that is preheated to 200 °C. The diameter of the as-cast cylindrical ingot is 80 mm. For the sake of convenience, the designed four alloys are denoted as 9Gd, 9Gd-1Y, 9Gd-3Y, and 9Gd-5Y, respectively. Table 1 shows the actual chemical compositions of the Mg-9Gd-*x*Y-0.5Zr alloys.

The microstructure is examined using scanning electron microscopy (SEM, TESCAN VEGA 3 LMH SEM, Tokyo, Japan). The second phases are analyzed by SEM equipped with a backscattered electron spectrometer (BSE) and energy-dispersive spectrometer (EDS) detector. The grain size of alloys with different Y contents is statistically analyzed using electron backscatter diffraction (EBSD, JEOL JSM-7800 F, Tokyo, Japan). The phase constitutions are detected by an X-ray diffractometer (XRD, Rigaku SmartLab, Japan). The microstructure is analyzed in depth using transmission electron microscopy (TEM, FEI Talos F200X, Thermo Fisher Scientific, Waltham, MA, USA). The area fraction of the second phase is evaluated using Image-Pro Plus 6.0 software. The oxidation tendency of different elements is calculated by HSC Chemistry 6.0 software.

**Figure 1:**
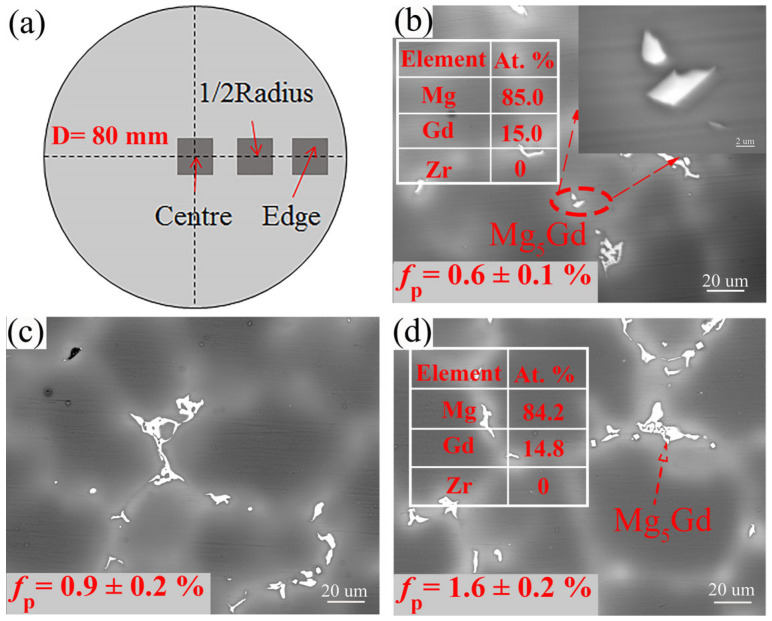
BSE-SEM images of the 9Gd alloy: (**a**) Schematic diagram of sampling location, (**b**) edge, (**c**) 1/2 radius, and (**d**), center.

As shown in Figure 1a, to ensure the consistency of the data, the observation location of the microstructure for the alloys is all at the 1/2 radius. The universal testing machine is CMT6305-300 KN (SUST, Shenzhen, China), and the strain rate is 1 mm/min. The tensile specimens (18 mm × 4 mm × 2.2 mm) are cut on the 1/2 radius of the cylindrical ingot, each sample is measured 3 times, and an error bar is added.

## 3. Results

### 3.1. Microstructure Evolution

**Figure 2:**
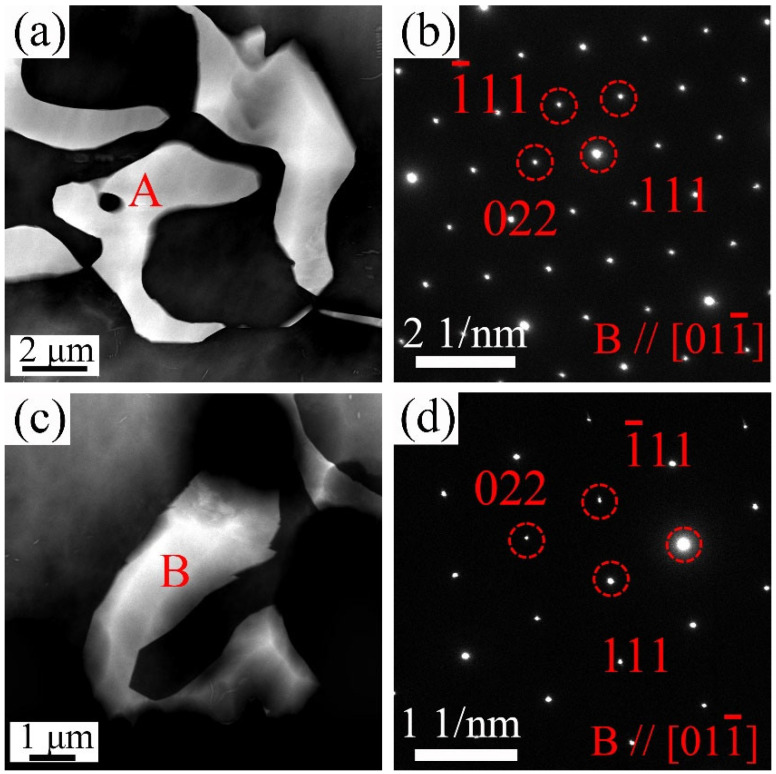
Microstructure characterization of the 9Gd alloy: (**a**,**c**) HAADF-STEM images, (**b**,**d**) SAED patterns (B//[01$\bar{1}$]_α_) of regions ‘A’ and ‘B’ in (**a**,**c**), respectively.

Figure 1b–d shows the BSE-SEM images of the 9Gd alloy at the edge, 1/2 radius, and center. The second phase is mainly Mg_5_Gd, which have various shapes and mainly appear as blocky and skeletal. The type of the second phase in different regions remains essentially unchanged. The area fraction of the Mg_5_Gd increases with the decrease in cooling rate, e.g., increasing from 0.6 ± 0.1% at the edge to 1.6 ± 0.2% at the center, indicating that the precipitation and growth of the Mg_5_Gd become easier. Figure 2 shows the HAADF-STEM images of the Mg_5_Gd with different shapes in the 9Gd alloy. Figure 2b,d shows the selected area electron diffraction (SAED) patterns of regions ‘A’ and ‘B’ in Figure 2a,c, and the HAADF-STEM images indicate that the second phases with different morphologies are both Mg_5_Gd.

**Figure 3:**
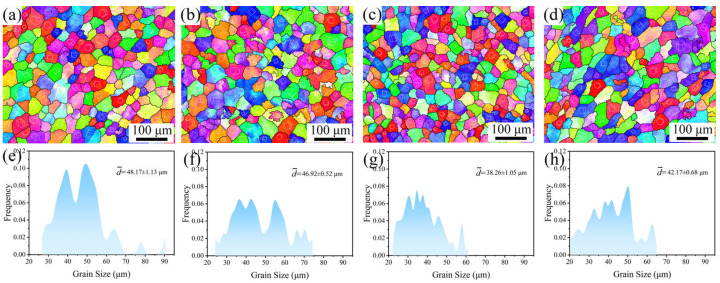
IPF images of alloys with different Y content at 1/2 radius: (**a**,**e**) 9Gd, (**b**,**f**) 9Gd-1Y, (**c**,**g**) 9Gd-3Y, (**d**,**h**) 9Gd-5Y.

**Figure 4:**
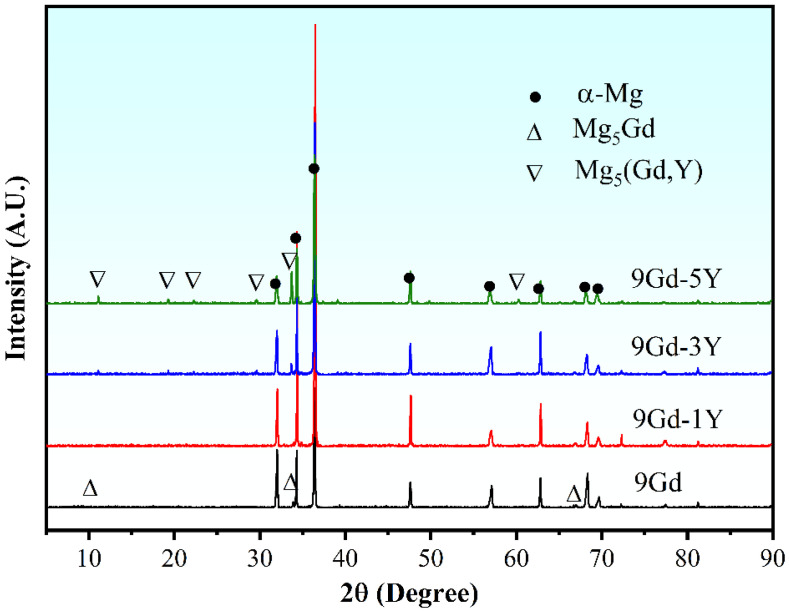
XRD patterns of the alloys with different Y content.

Figure 3 shows the EBSD results with different Y contents at 1/2 radius. The grain size of α-Mg decreases from 48.17 ± 1.13 μm to 38.26 ± 1.05 μm as the Y content increases. The reasons for changes in grain size will be explained in detail in Section 4.1. Appropriate Y content reduces the grain size but does not change the distribution and shape. Figure 4 shows the XRD pattern of the alloys with different Y contents. The second phase in the 9Gd alloy is Mg_5_Gd, which is consistent with those observed in Figure 1 and Figure 2. The type of the second phase changes to Mg_5_(Gd, Y) with the addition of Y, and the peak intensity increases with the increase in Y content.

**Figure 5:**
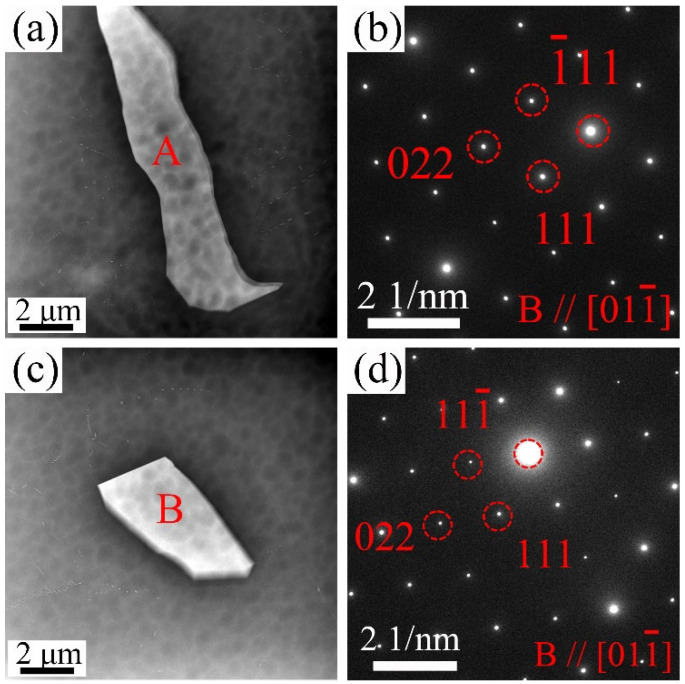
HAADF-STEM images and SAED patterns of the 9Gd-1Y alloy: (**a**,**c**) HAADF-STEM of second phases with different morphologies, (**b**,**d**) SAED patterns (B//[01$\bar{1}$]_α_) of regions ‘A’ and ‘B’ in (**a**,**c**), respectively.

**Figure 6:**
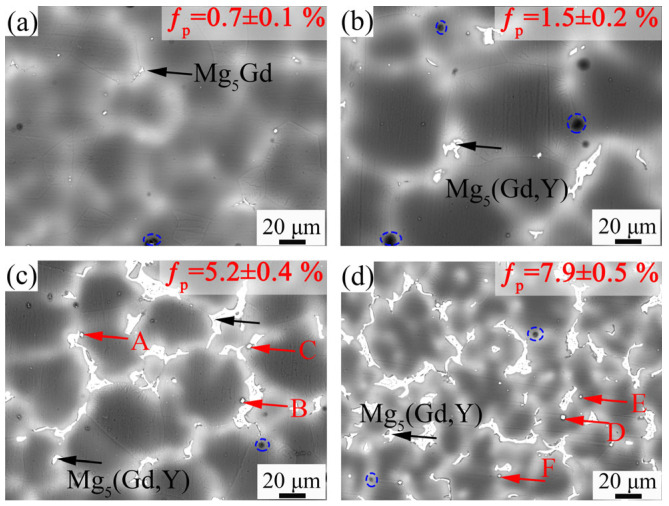
Distribution and morphology of the second phases in alloys with different Y contents: (**a**) 9Gd, (**b**) 9Gd-1Y, (**c**) 9Gd-3Y, (**d**) 9Gd-5Y, blue dashed circles indicate the pores in alloys.

Figure 5 shows the HAADF-STEM images of the second phase with different shapes in the 9Gd-1Y alloy. Figure 5b,d are the SAED patterns of regions ‘A’ and ‘B’ in Figure 5a,c, respectively, with the crystal zone axes B//[01$\bar{1}$]. The HAADF-STEM image indicates that the second phases with different morphologies are both Mg_5_(Gd,Y). Figure 6 shows the BSE-SEM images of the alloy at 1/2 radius. The second phase area fractions of the alloys with different Y contents are 0.7 ± 0.1%, 1.5 ± 0.2%, 5.2 ± 0.4%, and 7.9 ± 0.5%, respectively. In the 9Gd alloy, blocky Mg_5_Gd is mainly distributed at the grain boundary. With the increase in Y content, a large amount of Mg_5_(Gd, Y) appears at the grain boundaries, and the discontinuous intermetallic phase Mg_5_(Gd, Y) connects and grows. Additionally, in Figure 6c,d, fine-sized cubic phases, different from the blocky Mg_5_(Gd,Y), appear in the α-Mg matrix of the 9Gd-3Y and 9Gd-5Y, and the cubic phases (marked by red arrows) are marked as A, B, C, D, E, and F.

**Figure 7:**
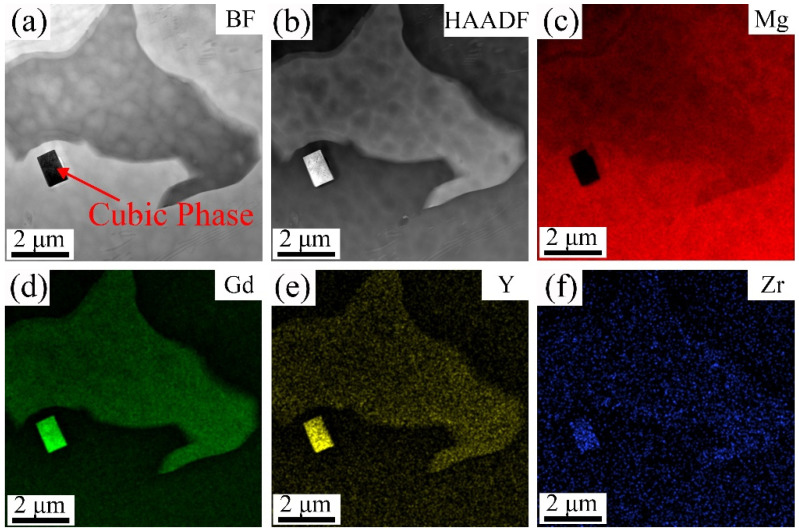
(**a**) TEM BF and (**b**) HAADF-STEM images of the 9Gd-3Y alloy: (**c**) mapping distribution of Mg, (**d**) mapping distribution of Gd, (**e**) mapping distribution of Y, (**f**) mapping distribution of Zr.

**Figure 8:**
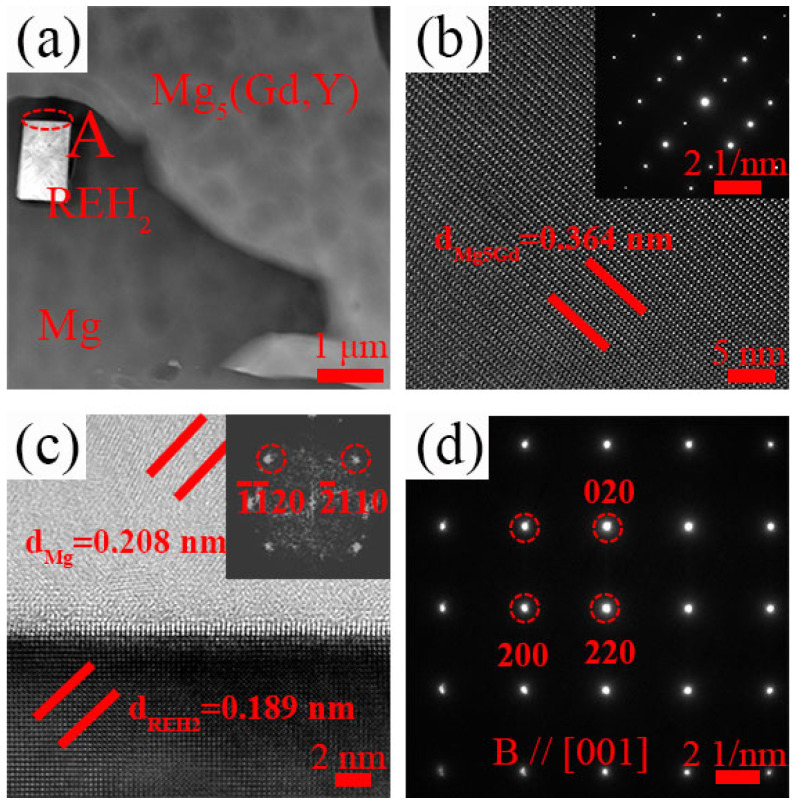
Microscopic characterization of the 9Gd-3Y alloy: (**a**) HAADF-STEM image, (**b**) HRTEM image of the Mg5(Gd,Y) phase, (**c**) HRTEM image of the Mg and REH_2_ phases in region ‘A’ of Figure 8a, (**d**) SAED pattern of the REH_2_ phase, with the zone axis B//[001].

To determine the element types in the cubic phase of Figure 6c (marked as A, B, and C), Figure 7a,b show the TEM BF and HAADF-STEM images of the 9Gd-3Y alloy, and Figure 7c–f show the mapping distribution of different elements. The Gd and Y are significantly enriched in the cubic phase, while Mg shows almost no enrichment. To further investigate the type of cubic phase, TEM is used to deeply characterize the microstructure of the 9Gd-3Y alloy. Figure 8a presents the HAADF-STEM characterization results of the Mg matrix and Mg_5_(Gd,Y). Figure 8b shows an HRTEM image of Mg_5_(Gd,Y) in Figure 8a. Figure 8c shows the HRTEM result of region ‘A’ in Figure 8a. Figure 8d is the SAED pattern of the cubic phase, where the crystal zone axis B is parallel to [001]. The results indicate that the cubic phase is REH_2_, in Figure 8c, d_Mg_ and d_REH2_ are 0.208 nm and 0.189 nm, respectively; the interface mismatch is 9.13%, and the crystallographic orientation relationship between REH_2_ and Mg is (220)_REH2_//(11$\bar{2}$0)_Mg_. The H atoms in the water vapor enter the alloy during the casting process; the electronegativity of Mg is higher than RE, resulting in a higher tendency for RE to bind with H and form REH_2_ [33,34,35].

**Figure 9:**
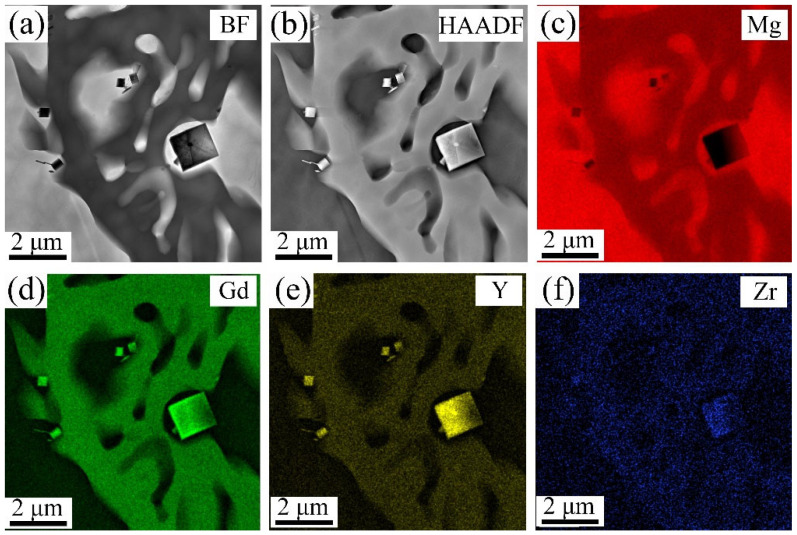
(**a**) TEM BF and (**b**) HAADF-STEM images of the 9Gd-5Y alloy, (**c**) mapping distribution of Mg, (**d**) mapping distribution of Gd, (**e**) mapping distribution of Y, (**f**) mapping distribution of Zr.

**Figure 10:**
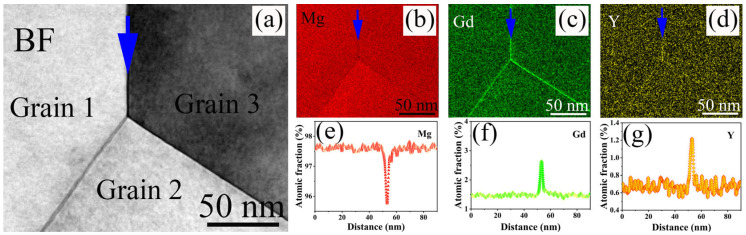
TEM images of element segregation at the grain boundary: (**a**) BF image, (**b**,**e**) Mg segregation result, (**c**,**f**) Gd segregation result, (**d**,**g**) Y segregation result.

The same phenomenon is also present in the 9Gd-5Y alloy. In Figure 9, the REH_2_ phase is densely distributed around the Mg_5_(Gd,Y), and the compositional mapping indicates that Gd and Y elements are enriched. In Figure 10, the Y element exhibits a strong tendency for segregation at grain boundaries, leading to the co-segregation of Gd and Y. This leads to a significant solute drag pressure (P*_d_*) [36], which reduces the grain boundary mobility rate, refines the grain size, and enhances the strength of the alloy. Additionally, the segregation of elements at grain boundaries may decrease the grain boundary energy [37] and pin the dislocation segments emanating from the grain boundaries [38], contributing to an increase in the yield strength of the alloy.

### 3.2. Tensile Mechanical Properties

**Figure 11:**
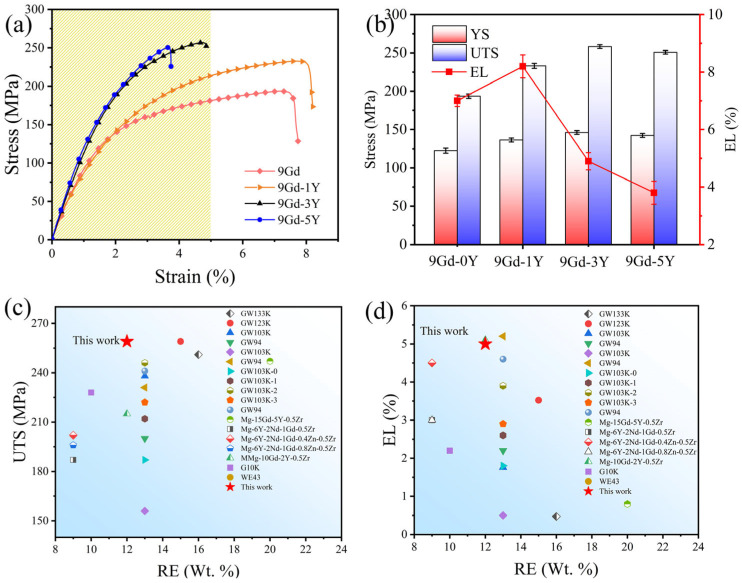
(**a**) Tensile strain-stress curves and (**b**) tensile properties of the alloys with different Y contents, (**c**,**d**) comparison of UTS and EL of between the 9Gd-3Y and previously reported Mg-RE alloys [30,31,32,39,40,41,42,43,44,45,46].

Figure 11a,b show the stress-strain curves and the tensile properties. The UTS, YS, and elongation (EL) of the 9Gd alloy are 193.5 MPa, 122.6 MPa, and 7.0%, respectively. The strength of the alloy increases when the Y content is 1 wt.%, and the elongation does not decrease but instead increases to 8.2%. The optimal comprehensive performance is the 9Gd-3Y alloy, with the UTS and YS being 258.2 MPa (33.4%) and 146.2 MPa (19.2%), respectively. By comparing the properties of 9Gd-3Y with previously reported Mg-RE alloys, in Figure 11c,d, the 9Gd-3Y has high strength and good ductility. Specifically, compared to GW94 [32], Mg-6Y-2Nd-1Gd-0.5Zr [39], GW103K-2 [40], and Mg-15Gd-5Y-0.5Zr [30], UTS increased by 59 MPa, 72 MPa, 13 MPa, and 12 MPa, respectively, and EL increased by 2.7%, 1.9%, 1.0%, and 4.1%, respectively. The 9Gd-3Y exhibits low RE content and superior performance, presenting significant economic advantages. The tensile strength and elongation of the 9Gd-5Y both decrease compared to the 9Gd-3Y, and the reasons will be discussed in Section 4.1.

**Figure 12:**
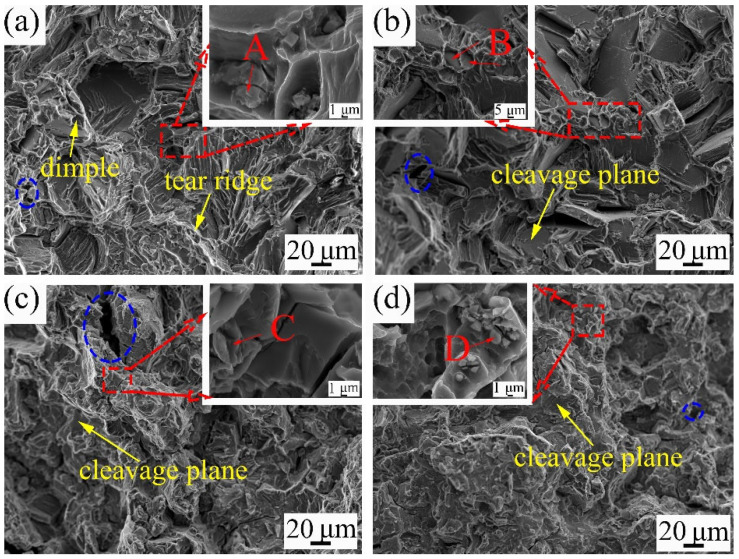
Tensile fracture surface morphologies of the alloys with different Y contents: (**a**) 9Gd, (**b**) 9Gd-1Y, (**c**) 9Gd-3Y, (**d**) 9Gd-5Y.

The fracture features of the alloys with different Y contents are shown in Figure 12. Table 2 shows the EDS analysis of the specially marked points on the fracture surface. The increase in Y content significantly affects the fracture behavior. In the 9Gd alloy, the fracture surface exhibits numerous dimples and tear ridges, along with bits of cleavage planes. Based on EDS results and previous studies [47], the phase denoted as ‘A’ is rich in Zr regions. In Figure 12b, when the Y content is 1 wt.%, the number of dimples and tear ridges on the fracture surface increases. The marked points ‘B’ and ‘C’ are both α-Mg, but they exhibit different morphological characteristics due to the decrease in ductility. In Figure 12d, the fracture surface becomes smoother and flatter, and the fracture mechanism changes from ductile fracture to brittle fracture, and the marked point ‘D’ is REH_2_. The area marked as the blue dashed ellipse indicates the pores.

**Figure 13:**
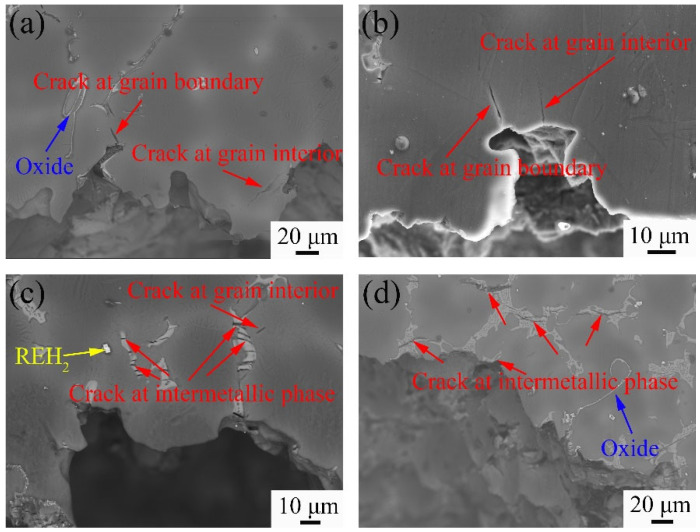
Fracture morphology of as-cast alloys with different Y contents on the longitudinal section (**a**) 9Gd, (**b**) 9Gd-1Y, (**c**) 9Gd-3Y, (**d**) 9Gd-5Y.

The fracture morphology parallel to the tensile direction is shown in Figure 13. When the Y content increases to 1 wt.%, the cracks occur in both the grain interior and at grain boundaries, resulting in intergranular and transgranular fracture. On the fracture surface of the 9Gd-3Y, the cracks begin to appear in the Mg_5_(Gd,Y), with a small number of cracks appearing in the matrix. The fracture mode is mainly intergranular fracture, supplemented by transgranular fracture. As the Y content is 5 wt.%, excessive Y promotes the growth and interconnection of the Mg_5_(Gd,Y), and cracks are easily generated and propagated inside Mg_5_(Gd,Y). The Mg_5_(Gd,Y) connect to each other and act as crack nucleation points and crack propagation paths, resulting in brittle fracture and poor performance. This is the reason why the comprehensive performance of the 9Gd-5Y is lower than that of the 9Gd-3Y. In Figure 13, the presence of oxides and REH_2_ is observed; however, cracks did not grow and propagate along the oxides and REH_2_ due to the low content and thus will not cause damage to the ductility.

**Figure 14:**
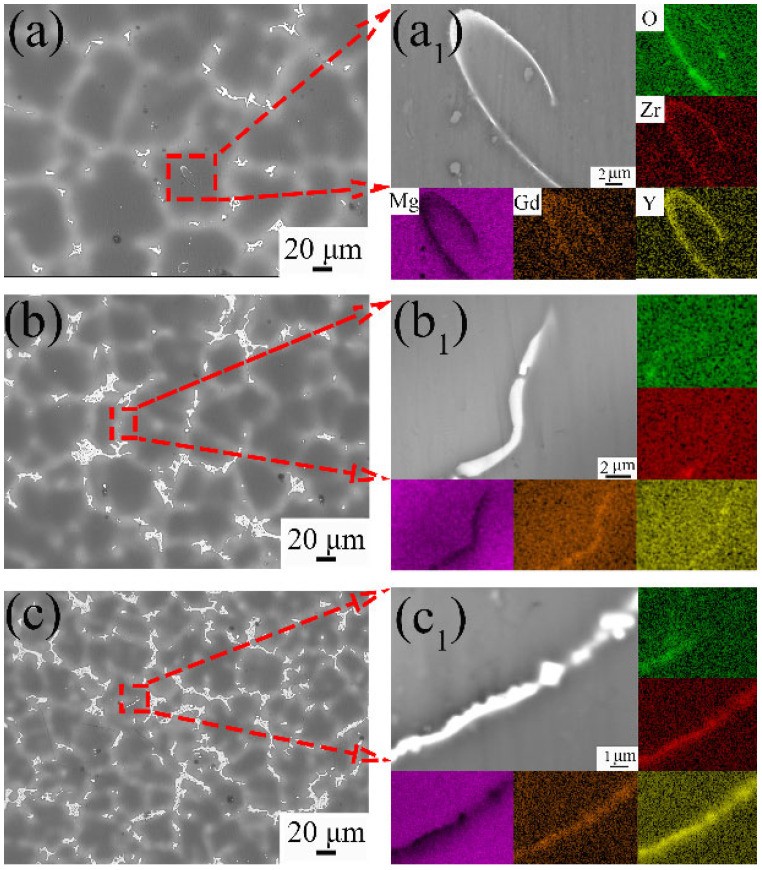
SEM images and EDS mappings of as-cast alloys with different Y contents: (**a**,**a_1_**) 9Gd-1Y, (**b**,**b_1_**) 9Gd-3Y, (**c**,**c_1_**) 9Gd-5Y.

**Figure 15:**
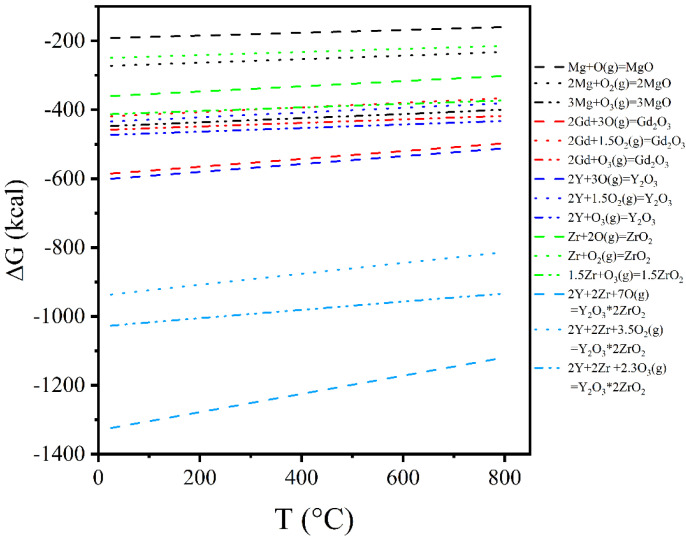
Oxidation tendency of different elements.

Figure 14 shows the SEM images and EDS mappings of the alloys with different Y contents. The oxidation of elements (Gd, Y, and Zr) occurs during the formation of Mg_5_(Gd,Y). To further evaluate the oxidation tendency, the reaction tendencies between different elements and oxygen atoms are calculated by HSC chemistry software, as shown in Figure 15. The Gibbs free energy for the Y reacting with oxygen atoms is the lowest, followed by Gd and Zr, and finally Mg, i.e., the reaction tendency is Y > Gd > Zr > Mg. The calculation results are consistent with the EDS mapping results.

## 4. Discussion

### 4.1. Effect of Y Content on Microstructure of the Mg-Gd-Y-Zr Alloys

As shown in Figure 1 and Figure 2, the shape and area fraction of the Mg_5_Gd will change at different positions. The decrease in the cooling rate leads to an increase in the area fraction of the Mg_5_Gd from 0.6 ± 0.1% to 1.6 ± 0.2% at different positions in the 9Gd alloy. As the Y content increases, the area fraction of the Mg_5_(Gd,Y) increases from 0.7 ± 0.1% to 7.9 ± 0.5%. In Figure 10, the co-segregation of Y elements reduces the grain boundary migration rate, which refines the grain size, and the grain size at 1/2 radius decreases from 48.17 μm to 38.26 μm. Additionally, the driving force for the formation of Mg_5_(Gd,Y) is enhanced with an increase in Y content; the increase in the area fraction of the Mg_5_(Gd,Y) at the grain boundaries also promotes a decrease in grain size. However, extensive Mg_5_(Gd,Y) is formed as the Y content exceeds the eutectic point. The separated intermetallic Mg_5_(Gd,Y) phases interconnect with each other, forming large skeletal Mg_5_(Gd,Y) in the 9Gd-5Y alloy, which will deteriorate the alloy properties. To avoid the formation of an excessively large second phase, an appropriate Y content is crucial. In addition, methods such as increasing the cooling rate, multi-process synergistic treatment (e.g., solution treatment + pre-deformation + aging treatment), friction stir processing (FSP), and extrusion deformation can break up the second phase and prevent excessive interconnection. In the 9Gd-5Y alloy, the Mg_5_(Gd,Y) phases grow along the grain boundaries, consuming the Y element, and there is no significant change in the width direction, so the change in grain size is not significant.

As shown in Figure 8 and Figure 9, the cubic phase REH_2_ is present in 9Gd-3Y and 9Gd-5Y. Nie et al. [40] suggested that the decomposition of Mg-RE phases by H during solidification may promote the formation of REH_2_, the REH_2_ phase has an fcc crystal structure with a = ~0.55 nm. The formation of REH_2_ will deteriorate the mechanical properties, especially EL [35]. However, as seen in Figure 13, the fracture of the as-cast alloy is primarily due to the extensive formation of cracks in the Mg_5_(Gd,Y), the REH_2_ phase is not the nucleation point for cracks. In Figure 8, d_Mg_ and d_REH2_ are 0.208 nm and 0.189 nm, respectively, with an interface mismatch of 9.13%, and there exists a crystallographic orientation relationship of (220)_REH2_//(11$\bar{2}$0)_Mg_. Therefore, REH_2_ in the as-cast Mg-Gd-Y-Zr alloy did not deteriorate the ductility, which is different from the conclusion in heat-treated alloys [34,35].

### 4.2. Effect of Y Content on Mechanical Properties of the Mg-Gd-Y-Zr Alloys

As shown in Figure 11, with the increase in Y content, the mechanical properties first increase and then decrease, i.e., obtaining the optimal mechanical properties in the 9Gd-3Y. When the Y content is low, the strengthening effect of Mg_5_(Gd,Y), with smaller grain size and area fraction, is inherently weaker. When the Y content is high, the excess Y promotes the growth and interconnection of the Mg_5_(Gd,Y). The strain incompatibility between the α-Mg matrix and the Mg_5_(Gd,Y) leads to stress concentration during the loading process, serving as crack nucleation points and propagation paths; thereby, the fracture mechanism of the 9Gd-5Y is transformed into intergranular fracture, deteriorating ductility and making the comprehensive performance of the 9Gd-5Y lower than that of the 9Gd-3Y. Additionally, the 9Gd-3Y alloy has the smallest grain size and can improve alloy properties through grain boundary strengthening.

### 4.3. Strengthening Mechanism of Mg-Gd-Y-Zr Alloy

The YS increased from 122.6 MPa in 9Gd to 146.2 MPa in 9Gd-3Y. The ∆*σ_YS_* can be explained from the perspectives of grain boundary strengthening (*σ_G_*), solid solution strengthening (*σ_S_*), and precipitation strengthening (*σ_P_*) [48,49], i.e.,

(1) $$∆{\sigma_{YS}=\sigma }_{YS(9Gd-3Y)}-\sigma_{YS(9Gd)}={∆\sigma }_{G}+{∆\sigma }_{S}+{∆\sigma }_{P}$$

where Δ*σ_G_*, Δ*σ_S_*, and Δ*σ_P_* represent the contributions of grain boundary strengthening, solid solution strengthening, and precipitation strengthening, respectively.

With the Y content increases, the grain size decreases from 48.17 μm to 38.26 μm. This phenomenon conforms to the Hall-Petch equation [50]:

(2) $$\sigma_{G}=\sigma_{0}+kd^{-1/2}$$

where *σ*_0_ and *k* are 11 MPa and 164 MPa μm^1/2^, respectively [7].

The contribution of adding Y to grain boundary strengthening can be expressed as:

(3) $$∆\sigma_{G}=k({d_{\left(9Gd-3Y\right)}}^{-\frac{1}{2}}-{d_{\left(9Gd\right)}}^{-\frac{1}{2}})$$

The calculated value Δ*σ_G_* is 3.3 MPa.

The solid solution strengthening of the 9Gd-3Y alloy can be approximated by the following relationship [51]:

(4) $$\sigma_{S}=\sum k_{i}c_{i}^{n}$$

where *n* is a constant, *k_i_* and *c_i_* are the relevant factor and solubility of solute *i*, respectively, and the solid solution strengthening contribution of adding Y can be assessed by the following relationship:

(5) $${∆\sigma }_{S}=k_{0.6}c_{0.6}^{n}$$

where *n* and *k_9Gd-3Y_* are 1/2 and 737 MPa (at%)^−1/2^, respectively [52]. According to the EDS results, the Y element content in different α-Mg matrixes is 0.13, 0.08, and 0.11 at. %, respectively, and the solubility of Y (*c*_0.6_) in the 9Gd-3Y alloy is approximately 0.11 at. %, and Δ*σ_S_* is calculated as 24.4 MPa.

The strengthening effect of the equilibrium β (Mg_5_RE) phase is calculated using the following equation [53,54]:

(6) $$\sigma_{P}=\frac{0.19MGb}{\lambda }ln\frac{0.08r}{b}$$

where *M*, *G*, λ, *r*, and *b* represent the Taylor factor, shear modulus, the distance of the β phases, the diameter of the β phases, and the burgers vector, respectively. The values of *M*, *G*, and *b* are taken as 2.5, 16,600 MPa, and 0.32 nm, respectively. The *r* of the particles in alloys with Y content of 0 and 3 wt.% are 4.73 µm and 9.57 µm, with λ of 33.1 µm and 8.63 µm, respectively. Therefore, Δ*σ_P_* is calculated to be approximately 1.8 MPa. 

The combined strengthening calculation results are shown in Table 3, and the main strengthening mechanism of the 9Gd-3Y is solid solution strengthening. The increase in YS is 23.6 MPa as the Y content increases to 3 wt.%, while the calculated strength difference is 29.5 MPa. The main reasons why the calculated values are slightly higher than the actual values include experimental errors and defects such as pores.

## 5. Conclusions

The effect of Y content on the microstructure and mechanical properties of the as-cast Mg-9Gd-*x*Y-0.5Zr alloys is investigated. A non-monotonous change in mechanical properties with the change in Y content and appropriate addition of Y can achieve the optimal comprehensive properties. The findings are summarized as follows:

(1) The 9Gd-3Y alloy exhibits the best mechanical properties with the increase in Y content. Compared with the 9Gd alloy, the UTS and YS increased approximately 33.4% and 19.2%, respectively, and the main strengthening mechanism is solid solution strengthening. The 9Gd-3Y alloy, with low RE content, high performance, and low cost, has significant economic advantages.

(2) REH_2_ phase appears in the 9Gd-3Y and 9Gd-5Y, the crystallographic orientation relationship between REH_2_ and Mg is (220)_REH2_//(11$\bar{2}$0)_Mg_, and the interface mismatch is 9.13%. Oxides and REH_2_ do not have a deteriorating impact on ductility, and the oxidation tendency of the elements in the alloy is Y > Gd > Zr > Mg.

(3) Excessive Y promotes the growth and interconnection of the Mg_5_(Gd,Y), becoming the nucleation point of cracks and paths of crack propagation, leading to the deterioration in the performance of the 9Gd-5Y alloy.

## Figures and Tables

**Table 1:** The actual chemical compositions of the Mg-9Gd-*x*Y-0.5Zr alloys (wt.%).

Alloys	Gd	Y	Zr	Mg
Mg-9Gd-0.5Zr	8.99	-	0.51	Bal.
Mg-9Gd-1Y-0.5Zr	8.97	1.03	0.49	Bal.
Mg-9Gd-3Y-0.5Zr	8.96	2.99	0.48	Bal.
Mg-9Gd-5Y-0.5Zr	9.04	5.03	0.51	Bal.

**Table 2:** EDS results for all points in Figure 12 (at%).

Points	Mg	Gd	Y	Zr
A	75.3	9.0	-	15.7
B	98.2	1.4	0.3	0.1
C	94.0	3.9	1.5	0.6
D	36.3	23.1	40.5	0.1

**Table 3:** Experimental values and calculated values of ∆*σ_YS_*.

Experimental Value (MPa)	Calculated Value (MPa)			
${∆\sigma }_{YS}$	${∆\sigma }_{YS}$	${∆\sigma }_{G}$	${∆\sigma }_{S}$	${∆\sigma }_{P}$
23.6	29.5	3.3	24.4	1.8

## Data Availability

The original contributions presented in this study are included in the article. Further inquiries can be directed to the corresponding authors.
